# Efficacy of toxic sugar bait with ivermectin against two fly species (Calliphoridae and Sarcophagidae) of medical and veterinary importance

**DOI:** 10.1186/s13071-025-07145-8

**Published:** 2026-01-03

**Authors:** Jaziela de Arruda Mendonça, Henrique Rafael Pontes Ferreira, Simão Dias Vasconcelos, Maria Alice Varjal de Melo-Santos

**Affiliations:** 1 Departament of Entomology, Aggeu Magalhães Institut, FIOCRUZ, Avenida Moraes Rego s/n, Campus da Universidade Federal de Pernambuco, Cidade Universitária, Recife, Pernambuco CEP 50670420 Brazil; 2 Laboratory of Insects of Forensic Importance, Universidade Federal de Pernambuco/UFPE, Recife, Pernambuco Brazil

**Keywords:** *Chrysomya megacephala*, *Peckia* (*Sarcodexia*) *lambens*, Synanthropic fly, Behavioural control, Sugar feeding

## Abstract

**Background:**

The use of toxic sugar bait with ivermectin (TSB_Ivermec_) has emerged as a promising strategy for controlling insects that feed on carbohydrates, particularly mosquito species, in areas endemic for arbovirus transmission. The aim of this study was to evaluate the effectiveness of TSB_Ivermec_ in controlling flies of medical and veterinary importance. We assessed the overall mortality of adults of two species, *Peckia* (*Sarcodexia*) *lambens* and *Chrysomya megacephala*, and the effect of different ivermectin concentrations on fly mortality.

**Methods:**

Concentrations of 0.012%, 0.025%, and 0.050% ivermectin diluted in 20% sucrose were tested on groups of 40 adult flies (1:1 male:female), aged 4–8 days. Exposure to TSB_Ivermec_ lasted for 6 h, and mortality was monitored for up to 96 h and compared to that of a control group with no ivermectin exposure.

**Results:**

Both species showed cumulative mortality of 100% at the highest concentration of ivermectin (0.050%), although *P.* (*S*.) *lambens* was more sensitive to the compound than *C. megacephala*, with most deaths occurring within the first 24 h. At lower concentrations, mortality ranged from 97 to 81% for *P.* (*S*.) *lambens* (at 0.025% and 0.012%, respectively), and 78–57% for *C. megacephala*. Mortality differed significantly between these species (*P* < 0.001), but not between the sexes (*P* = 0.8). TSB_Ivermec_ demonstrated high efficacy against both species under semi-field conditions.

**Conclusions:**

The results suggest that the 0.050% concentration of ivermectin, which is known to be effective against mosquitoes, is also optimal for the rapid population control of synanthropic flies, enabling its integrated use in highly infested or vulnerable environments, such as health care facilities.

**Graphical Abstract:**

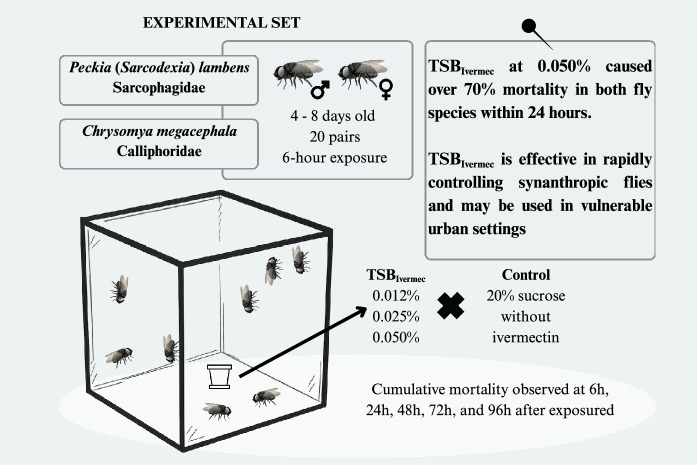

## Background

Tropical countries have a high diversity and abundance of species of the Muscomorpha (Diptera), which can act as both mechanical and biological vectors. According to recent research, Brazil ranks among the countries with the highest prevalence of parasites transmitted by non-biting Diptera [1]. Blow flies (Calliphoridae) and flesh flies (Sarcophagidae) transmit, biologically or mechanically, a wide diversity of pathogens and parasites, including bacteria (e.g. *Enterococcus, Staphylococcus, Escherichia, Proteus, Enterobacter, Klebsiella, Morganella, Bordetella, Serratia, Plesiomonas, Wohlfahrtiimonas* [2]), nematodes (e.g. *Ascaris, Trichuris*) [3]) and protozoans (e.g. *Sarcocystis*, *Toxoplasma*, *Isospora*, *Giardia*, *Entamoeba*, *Endolimax*, *Trichomonas*, *Hammondia*, *Cryptosporidium* [4]).

Flies function as reservoirs, transferring pathogens through direct contact or regurgitation/defecation on surfaces [5]. Thus, flies help to distribute multi-drug-resistant bacteria and their drug-resistance genes in certain ecosystems and habitats [6], which is enhanced by their specialized feeding on decaying organic matter. Furthermore, some flies cause myiasis in humans and non-human animals, especially in South America [7]. Patients may suffer from serious complications, such as secondary infections, recurrent infestations, chronic pain, amputation of limbs, and impaired quality of life [8].

Methods for the control of medically important flies rely on a wide spectrum of chemical pesticides with high residual power. The disadvantages of these are well known and include physiological or behavioural resistance, deleterious effects on non-target organisms, such as pollinators, and, importantly, risks to human health, which can even lead to death [9, 10]. In healthcare units, spraying pesticides is impractical, which has inspired the development of novel approaches.

A well-known strategy for the control of insects consists of the use of a toxic sugar bait (TSB). This strategy is not new, but its use has increased in the last decades. For the Diptera, TSBs comprising different classes of toxic substances have been successfully used to suppress populations of *Phlebotomus* [11], *Aedes* [12], *Culex* [13], and *Anopheles* [14].

As part of a project for the multiple-strategy control of populations of Calliphoridae and Sarcophagidae in public healthcare facilities, we developed a TSB for evaluation in hospitals in rural zones of northeastern Brazil, where there is a high risk of myiasis. As the toxic compound, we selected ivermectin (C_48_H_74_O_14_), a common parasiticide used in cattle due to its low cost and efficacy against endo- and ectoparasites [15]. Ivermectin binds to glutamate-gated and gamma-aminobutyric acid receptors, modifying membrane permeability to chloride ions in invertebrates, and thus affects functions of the nervous and muscular systems. These effects cause paralysis and death [16]. Ivermectin is also used to control the larvae of species of the Calliphoridae that cause myiasis, e.g. *Cochliomyia hominivorax* (Coquerel) and *Chrysomya* spp. [17].

The objective of this study was to evaluate the efficacy of sugar bait with ivermectin (TSB_Ivermec_) as a model for the control of adult flies of medical and veterinary importance. Specifically, we aimed to assess (1) the overall mortality of *Chrysomya megacephala* (Fabricius) and *Peckia (Sarcodexia) lambens* (Wiedemann); (2) the mean time to death of the adult flies; (3) the effect of different concentrations of ivermectin on the mortality and the time to death; (4) the sex of the dead/surviving individuals.

## Methods

### Insect-rearing and experimental area

The specimens used in the experiments were derived from a field-collected subpopulation from Santa Cruz do Capibaribe, Pernambuco, northeastern Brazil. The flies were maintained under laboratory conditions—temperature 25 ± 2 °C, relative humidity 70 ± 5% and a photoperiod of 12:12 h (light:dark) for three successive generations. Adults of each species were reared separately in plastic cages (40 × 60 × 40 cm), and were provided with a 50:50 sucrose-water solution *ad libitum*, and ground beef as a substrate for oviposition (*C. megacephala*) or larviposition [*P.* (*S*.) *lambens*], in accordance with Ferreira et al. [18]. Each cage contained 30 pairs of insects. These were monitored twice a day and the substrates containing eggs/larvae removed to obtain the adult flies for the tests carried out under semi-field conditions. When substrate was removed, it was replaced with fresh substrate.

All trials with TSB_Ivermec_ were conducted in the experimental semi-field area, a 24-m^2^ outdoor space protected by a roof from the rain and exposure to direct sunlight. This area is located at the Instituto Aggeu Magalhães/IAM (FIOCRUZ, Pernambuco), Recife (8.0522°S, 34.9286°W), northeastern Brazil. Temperature and relative humidity were monitored during the experiments.

### Design of the experiments 

The objectives of the tests were as follows: to prove the efficacy of ivermectin to kill adult flies; to determine the optimal recommended concentration of TSB_Ivermec_ for use in the field; and to evaluate the effect of sex and body size on fly mortality.

At 4 to 8 days post-emergence, adults of *C. megacephala* and *P.* (*S.*) *lambens* originating from the same laboratory-reared generation were separated into groups of 40 couples and introduced into the experimental cages for each treatment. The cages were constructed of metal and fine mesh (20 × 40 × 20 cm). There were three replicates, comprising the same number of flies for each species, for each tested concentration of TSB_Ivermec_.

We used ivermectin in its commercial liquid formulation (JA Saúde Animal^®^) containing 1% (10,000 p.p.m.) of the active principle from a 500-mL stock solution. To achieve experimental concentrations of 0.012% (low), 0.025% (intermediate), and 0.050% (high) ivermectin, we performed a serial dilution in a 20% sucrose solution (weight/volume), with each dilution adjusted to a final volume of 100 mL. We prepared a 0.1% intermediate solution by mixing 10 mL of the stock solution with 90 mL of 20% sucrose (1:10 dilution). From this, we devised the target concentrations. The 20% sucrose solution served as both a diluent and an attractant to ensure the uniform distribution and palatability of the toxic suspension.

The *C. megacephala* and *P.* (*S.*) *lambens* selected for the experiments underwent acclimatization and were starved for 1 h before the TSB_Ivermec_ trials. Each experimental cage contained a feeding substrate consisting of a 0.6 g cotton pad soaked in 20 mL of the control or one of the treatments (low, intermediate, or high concentration of ivermectin), which was placed inside a transparent 100-mL plastic container. The control groups were subjected to the same experimental conditions but the adults were fed only with the sugar solution. We used the same batch of the product, a fresh suspension of TSB_Ivermec_, and a new cohort of flies, in three independent tests on different days.

The tested species were exposed to all treatments simultaneously in the experimental semi-field area, where the mean temperature was 31.4 ºC (23.8–36.6 ºC) and the relative humidity was 54.5% (43.5–90.2%).

As part of an ecologically sound strategy for use in inhabited areas, we opted for a short exposure period (6 h) to TSB_Ivermec_, which was applied only once, when the flies were released into the experimental cages. The aim of this procedure was to assess acute toxicity exposure, based on the possible scenario of brief contact in the field, particularly under conditions in which other carbohydrate sources compete with the TSB.

After 6 h of *ad libitum* feeding on the TSB_Ivermec_, the substrates were removed from all the cages and replaced with clean cotton containing only 20% sucrose solution.

Mortality was first assessed after 6 h exposure to TSB_Ivermec_ and subsequently at 24 h, 48 h, 72 h, and 96 h following TSB_Ivermec_ removal. The 24 h timepoint was established as a reference for evaluating the rapid lethality of the compound. At each observation timepoint, dead flies were counted and immediately removed from the cage, then sexed and preserved in 70% alcohol.

Furthermore, an additional batch of flies, comprising 100 adults of each species that had been reared in the laboratory and belonged to the same generation, underwent body length measurements to determine the mean size of each species. The specimens were positioned laterally and photographed under a stereomicroscope at ×40 magnification. The images were analysed using tpsDig246^®^ software, and the total body length, from the tip of the head to the end of the abdomen, was measured in millimetres.

### Statistical analysis

The dependent variables analysed were (1) mortality [proportion (%): number of dead insects/total individuals × 100]; (2) the time elapsed until death; (3) the survivorship pattern; and (4) the sex of dead/surviving individuals. The interaction between survival and TSB_Ivermec_ concentration was elucidated using a generalized linear model, with the coefficients revealing mortality patterns for each species.

To ensure analytical robustness, we first performed a Shapiro-Wilk test (*P* < 0.05) to assess data normality. Normally distributed data were analysed via ANOVA, followed by Tukey’s post hoc test, while non-normal data were evaluated using the Kruskal-Wallis test with Bonferroni correction. We applied a generalized linear model to assess the effects of TSB_Ivermec_ across concentrations, species, and sex on survival, and we also generated daily survival curves via the Kaplan-Meier method. Statistical significance was determined at *P* < 0.05 and the analyses were conducted using R software^®^ (4.4.1).

## Results

### TSB_Ivermec_ was lethal to the flies regardless of species or sex

**Fig. 1:**
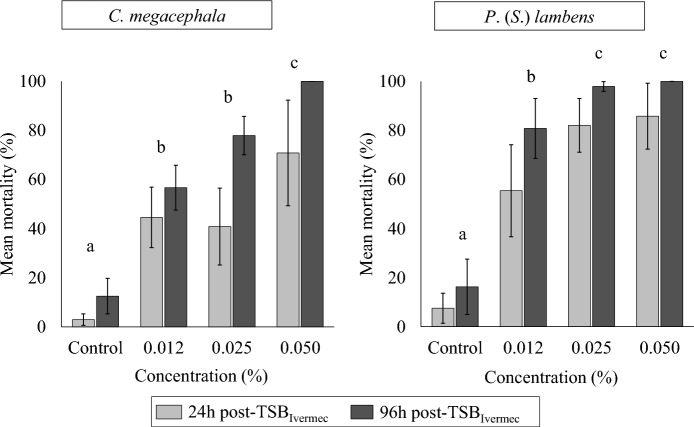
Mean mortality (%; ± SD) of adult flies at 24 and 96 h after exposure to different concentrations of toxic sugar bait with ivermectin (TSB_Ivermec_)

Exposure of both fly species to TSB_Ivermec_ resulted in a reduction in survival within a few hours, depending on the concentration. Within the first 6 h, the 0.050% formulation caused 30% mortality in *C. megacephala* and 23.3% in *P*. (*S*.) *lambens*, while at lower concentrations, mortality did not exceed 16%. Rapid action of TSB_Ivermec_ was demonstrated, with *P*. (*S*.) *lambens* showing mortality rates above 80% in the first 24 h at concentrations of 0.025% (82%) and 0.050% (85.8%). For *C. megacephala*, mortality only exceeded 70% at the highest concentration evaluated (0.050%) (Fig. 1).

At 96 h, mortality of 100% was reached for both species at TSB_Ivermec_ 0.050%. For *P*. (*S*.) *lambens*, the intermediate dose (0.025%), for which the mortality was 97%, performed similarly to the highest dose (*Z* = 1.3,* df* = 3, *P* = 1). For *C. megacephala*, TSB_Ivermec_ 0.025% resulted in 80% mortality, which was significantly lower than that obtained at a dose of 0.050% (*Z* = 3.79,* df* = 3, *P* < 0.001).

At the lowest concentration (0.012%), *P*. (*S*.) *lambens* showed 55.4% mortality within the first 24 h and a cumulative mortality rate of 81% by the end of the experiment, which was significantly higher than that of the control (*Z* = 5.6,* df* = 3, *P* < 0.001). However, despite its high lethality, this dose was less effective than the higher doses. For *C. megacephala*, the cumulative mortalities of 56.6% and 80% at 0.012% and 0.025% TSB_Ivermec_, respectively, were statistically similar (*Z* = 1.27,* df* = 3, *P* = 1). The effect of TSB_Ivermec_ 0.012% was significantly greater than that of the control treatment (*Z* = 6.07,* df* = 3, *P* < 0.001).

The different TSB_Ivermec_ concentrations induced significantly distinct mortality rates between *C. megacephala* and *P*. (*S*.) *lambens* (*χ*^2^ = 11.6, *df* = 1,* P* < 0.001). In contrast, the sex of the specimens did not influence treatment response: males and females of both species showed similar mortality rates (*χ*^2^ = 0.03, *df* = 1,* P* = 0.80).

The body size of the two species differed significantly (*χ*^2^ = 43.65,* df* = 1, *P* < 0.001), with *C. megacephala* being larger (*x̅* = 8.32 ± 0.75 mm) than *P*. (*S*.) *lambens* (*x̅* = 7.70 ± 0.70 mm).

### Survival was inversely proportional to the concentration of TSB_Ivermec_

There was a negative and inversely proportional relationship between the concentration of ivermectin and survival probability, i.e. the higher the dose, the lower the survival rate for both *C. megacephala* (*β* = − 71.8, *Z* = − 31.23, *P* < 0.001) and *P*. (*S*.) *lambens* (*β* = − 76.53, *Z* = − 30.18, *P* < 0.001).

**Fig. 2:**
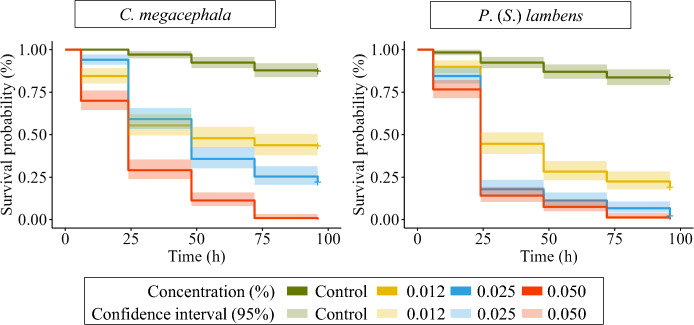
Survival probability of adult flies of *Chrysomya megacephala* and *Peckia* (*Sarcodexia*) *lambens* in response to different concentrations of TSB_Ivermec_ over the 96-h post-exposure period

Survival steadily declined over time across all concentrations, with the death of all individuals occurring within 72 h at the 0.050% dose for both species. At 96 h, the 0.025% concentration had led to the mortality of all *P*. (*S*.) *lambens*, whereas approximately 25% of *C. megacephala* individuals remained alive at this timepoint (Fig. 2).

Within the first 24 h, the 0.050% concentration caused a sharp reduction in survival for both species, with survival probabilities ranging between 15% and 40%. From that point onward, survival in this treatment continued to decline progressively over time, and became null within 96 h. In the control experiments, the survival probability remained high, and ranged from 85 to 90% at the end of the observation period.

## Discussion

Controlling medically important flies is a major challenge in the Neotropical region, especially because of poor hygiene, ineffective educational campaigns, and over-reliance on the spraying of broad-spectrum insecticides in several countries. We demonstrate here that TSB containing ivermectin caused high mortality in two species associated with myiasis and the transmission of pathogens to humans and other animals.

To the best of our knowledge, this study represents the first empirical test of TSB_Ivermec_ for the control of populations of blow flies and flesh flies. The results are encouraging. Mortality was high for *P. (S) lambens* under the three concentrations evaluated (0.012%, 0.025% and 0.050%), and surpassed 80.0% at the lowest concentration at 96 h post-exposure. In comparison, the mortality of *C. megacephala* was slightly lower under the low and intermediate concentrations.

Although 100% mortality was achieved within 96 h at the 0.050% concentration, mortality rates varied between the two species, highlighting clear differences in their susceptibility. Considering that the effectiveness of most insecticides depends on the size of the insect, we believe that the larger size of *C. megacephala* may have contributed to its (comparatively) lower mortality. However, differences in individual levels of tolerance to the ivermectin or the amount ingested by the flies cannot be ruled out.

The efficacy of TSB_Ivermec_ was further confirmed by the survival probability curve. A major drop in survival was observed for both species as early as 24 h post-exposure. The combination of high mortality and immediate death makes TSB_Ivermec_ an ideal candidate for the control of Muscomorpha, and corroborates a principle established 60 years ago. Lea [19] evaluated, under experimental conditions, the concept of adding insecticide to a sugary meal for the control of mosquitoes. However, studies undertaken in the field prioritized the suppression of populations of Culicidae, Psychodidae and Simuliidae [20], with varying degrees of success. Species of the Calliphoridae and Sarcophagidae have been previously neglected.

Flesh flies and blow flies can carry over 20 species of bacteria [21, 22]. Species of *Chrysomya* and *Peckia* cause myiasis in humans and other animals, especially in rural zones of Neotropical countries, yet their impact on human health suffers from marked under-reporting [7, 8]. The neglect of elderly/undernourished people increases the incidence of myiasis in Brazil, with reports of infestations with multiple fly species when treatment is not provided [8]. As vectors, their habit of feeding on decomposing animal matter and subsequently landing on human food aggravates the cycle of diseases that they carry, especially in Brazil, where over 27 million people do not have access to basic sanitation or sewage systems [23]. Thus, methods for fly control must be simple to carry out, inexpensive, easy to monitor, and suitable for combination with other techniques.

The successful suppression of populations of *Anopheles sergentii* (Theobald) and *Aedes caspius* (Pallas) in Israel was reported after the use of attractive toxic sugar bait containing the insecticide spinosad [24]. Other examples of species successfully suppressed with TSB include *Anopheles gambiae*, in Mali [25], *Culex quinquefasciatus* (Say) and *Aedes aegypti* (Linnaeus), in Morocco [26], and *Aedes albopictus* (Skuse), in the USA [27]. The use of TSB with or without attractants, such as fruit juice, has been documented in several countries. This tool, used alone or integrated with other strategies, has shown satisfactory results against mosquitoes such as *Aedes* [28, 29], *Culex* [13, 28] and *Anopheles* [24].

A recent initiative for mosquito control in Brazil demonstrated that 0.050% ivermectin, a dose also used in our study, caused > 90% mortality of adult *A. aegypti* and *C. quinquefasciatus* in the field [28, 29]; this shows that the same dose of ivermectin can kill both mosquitoes and flies. In the present study, short-term exposure to TSB_Ivermec_ (6 h) was sufficient to cause a notable rate of mortality in *P*. (*S*.) *lambens* and *C. megacephala* 24 h after ingestion, even at the lowest concentration tested (0.012%), suggesting that ivermectin may be used effectively in targeted control strategies.

However, despite its low cost and high efficacy, risks are associated with ivermectin, including its broad-spectrum effects, which can cause mortality in non-target organisms and its accumulation in the soil and aquatic environments [30]. To mitigate these potential impacts, it is recommended that TSB_Ivermec_ used for the treatment of endophilic insects searching for sources of carbohydrates in different types of households, hospitals and other public buildings should be applied at attractant stations in pre-determined locations [28, 29].

Most studies on the use of ivermectin for fly control have focused on indirect exposure, particularly through ingestion by the larvae of the faeces of animals previously treated with the drug, or for the treatment of myiasis. The reported effects include larval mortality, delayed development, and reduced adult emergence when larvae are the targets [8, 31, 32]. In contrast, this study addressed the direct ingestion by adult flies of the compound from ivermectin-laced bait. This strategy increases the success of controlling the flies by eliminating the adults before oviposition.

The search for efficacious and fast acting strategies for the control of flies in vulnerable places, such as hospitals, where the use of conventional insecticides is impractical, shows that TSB_Ivermec_ is a viable alternative as it can be strategically used in indoor areas only, with minimal risk to patients, leading to a reduction in cases of myiasis, pathogen transmission and contact with non-target species, such as bees and other pollinators which may be present around the periphery of health facilities and homes (Table 1).

**Table 1:** Some advantages, and limitations, of TSB_Ivermec_ for the control of species of the Muscomorpha of medical and parasitological importance

Advantages	Limitations
Its use is based on the active behaviour of vectors searching for carbohydrate sources Its preparation is easy Low cost Focal and practical indoor use Trained personnel are not required Easy to use together with other control methods, such as larvicidal products Fast acting with high mortality of blow flies and flesh flies It can be used in rural zones and in areas with poor sanitation Suitable for use during outbreaks of blow flies and flesh flies Effective in the control of other Diptera (e.g. mosquitoes) It can be used in environments where spraying insecticides is infeasible (e.g. hospitals, restaurants, daycare centres, schools)	Ivermectin has broad-spectrum activity, which can affect non-target organisms (e.g. pollinators) Ivermectin can accumulate in soil and freshwater ecosystems Baits need to be replaced regularly Community participation is required

 Based on the criteria evaluated in the present study, TSB_Ivermec_ proved to be a promising tool. In a sugar-based formulation it has increased selectivity for synanthropic species, and its use within buildings can potentially minimize its impacts on non-target organisms, as these are less likely to enter and thus encounter the bait when it is used in these environments [20, 25, 28, 29]. Moreover, its simple preparation and low cost provide support for its adoption in cities under financial constraints. Although further research is still needed, the method examined here demonstrated effectiveness, simplicity, and is considered safe and sustainable.

Interestingly, we observed that mortality did not differ between males and females of either species. In contrast, in a study undertaken by Chaaban et al. [33] on the susceptibility of *C. megacephala* and *Lucilia cuprina* (Wiedemann) to S,S'-bis(diisobutylphosphoryl)-1,3-propanedithiol, a more toxic effect was demonstrated for males than for females of *L. cuprina*, while for *C. megacephala,* the females were more sensitive than the males.

## Conclusions

This study evaluated the adulticidal effect of TSB_Ivermec_ on sarcosaprophagous flies under semi-field conditions and demonstrated the potential of this strategy as an efficient tool for insect control. In a country like Brazil with inequalities in public health coverage, the use of alternative control strategies designed to reduce populations of ectoparasites and vectors is essential. Although implementation of this type of strategy faces challenges, such as logistical difficulties, the need for staff training and continuous evaluation, we advocate for insect control with TSB_Ivermec_, firstly in controlled environments and later, if successful, in areas where funding for the protection of public health is limited.

## Data Availability

All data generated or analysed during this study are included in this published article.
